# Rapid dose-dependent Natural Killer (NK) cell modulation and cytokine responses following human rVSV-ZEBOV Ebolavirus vaccination

**DOI:** 10.1038/s41541-020-0179-4

**Published:** 2020-04-14

**Authors:** David Pejoski, Casimir de Rham, Paola Martinez-Murillo, Francesco Santoro, Floriane Auderset, Donata Medaglini, Gianni Pozzi, Maria Vono, Paul-Henri Lambert, Angela Huttner, Mariëlle C. Haks, Tom H. M. Ottenhoff, Jean Villard, Claire-Anne Siegrist, the VEBCON Consortium, Marylyn M. Addo, Selidji Todagbe Agnandji, Stephan Becker, Philip Bejon, Jessica S. Brosnahan, Patricia Fast, Angela Huttner, Verena Krähling, Marie-Paule Kieny, Peter G. Kremsner, Sanjeev Krishna, Olivier Lapujade, Vasee Moorthy, Patricia Njuguna, Barbara Savarese, Claire-Anne Siegrist, VSV-EBOVAC Consortium, Selidji Todagbe Agnandji, Rafi Ahmed, Jenna Anderson, Floriane Auderset, Philip Bejon, Luisa Borgianni, Jessica S. Brosnahan, Annalisa Ciabattini, Olivier Engler, Mariëlle C. Haks, Ali M. Harandi, Donald Gray Heppner, Alice Gerlini, Angela Huttner, Peter G. Kremsner, Paola Martinez-Murillo, Donata Medaglini, Thomas Monath, Francis Ndungu, Patricia Njuguna, Tom H. M. Ottenhoff, Mark Page, David Pejoski, Gianni Pozzi, Francesco Santoro, Claire-Anne Siegrist, VSV-EBOPLUS Consortium, Selidji Todagbe Agnandji, Jenna Anderson, Floriane Auderset, Luisa Borgianni, Annalisa Ciabattini, Sheri Dubey, Olivier Engler, José F. Fernandes, Mariëlle C. Haks, Ali M. Harandi, Alice Gerlini, Angela Huttner, Peter G. Kremsner, Paola Martinez-Murillo, Donata Medaglini, Thomas Monath, Helder Nakaya, Fiona O’Rourke, Tom H. M. Ottenhoff, David Pejoski, Gianni Pozzi, Sylvia Rothenberger, Francesco Santoro, Claire-Anne Siegrist

**Affiliations:** 1 Department of Pathology and Immunology, Faculty of Medicine, University of Geneva, Geneva, Switzerland; 2 World Health Organization Collaborating Centre for Vaccine Immunology, Faculty of Medicine, University of Geneva, Geneva, Switzerland; 3 Immunology and Transplantation Unit, Geneva University Hospitals, Geneva, Switzerland; 4 Department of Medical Biotechnologies, University of Siena, Siena, Italy; 5 Infectious Diseases Service, Geneva University Hospitals, Geneva, Switzerland; 6 Department of Infectious Diseases, Leiden University Medical Center, Leiden, The Netherlands; 7 University Medical Center Hamburg, Hamburg, Germany; 8 Centre de Recherches Médicales de Lambaréné, Lambaréné, Gabon; 9 Kenya Medical Research Institute, Kilifi, Kenya; 10 Institut für Tropenmedizin, Universitätsklinikum Tübingen, Tübingen, Germany; 11 World Health Organization, Geneva, Switzerland; 12 Institute of Virology, Marburg, Germany; 13 St George’s University of London, London, UK; 14 Emory Vaccine Centre, Atlanta, GA USA; 15 University of Gothenburg, Gothenburg, Sweden; 16 Sclavo Vaccines Association, Siena, Italy; 17 Spiez Laboratory, Spiez, Switzerland; 18 Crozet BioPharma, Devens, MA USA; 19 Newlink Genetics, Ames, IA USA; 20 Centre for Tropical Medicine and Global Health, Oxford University, Oxford, UK; 21 National Institute for Biological Standards and Control, Hertfordshire, UK; 22 Merck Research Laboratories, Merck and Co., Kenilworth, NJ USA; 23 Department of Clinical Analyses and Toxicology, University of Sao Paolo, Sao Paolo, Brazil

**Keywords:** Innate immunity, Vaccines, Live attenuated vaccines

## Abstract

The rVSV-ZEBOV Ebolavirus vaccine confers protection within days after immunization, suggesting the contribution of innate immune responses. We report modulation of rVSV-ZEBOV vaccinee blood CD56^+^ NK cell numbers, NKG2D or NKp30 surface receptor expression, Killer Immunoglobulin-like Receptor (KIR)^+^ cell percentages and NK-cell-related genes on day 1 post immunization. Inverse correlations existed between the concentration of several plasma cytokines and inhibitory KIR^+^ CD56^dim^ or cytokine-responsive CD56^bright^ NK cells. Thus, NK cells may contribute to the early protective efficacy of rVSV-ZEBOV in humans.

## Introduction

Ebolavirus continues to cause deadly epidemics. The live-attenuated rVSV-ZEBOV vectored vaccine has now been employed in over 255,000 people in “ring vaccination” trials in Africa^1,2^, mediating 97.5% protection if administered at least 10 days prior to contact with infected individuals, as well as partial protection for shorter periods between vaccination and exposure (https://www.who.int/csr/resources/publications/ebola/ebola-ring-vaccination-results-12-april-2019.pdf). Early protection is vital to interrupt viral outbreaks. As 2 weeks are required for ZEBOV glycoprotein (GP)-specific IgG antibodies to appear in the blood of most vaccinees^3^, and at least 3–7 days for IgM^4^, innate vaccine-elicited responses may contribute to the rapid protection.

NK cells have clear roles in antiviral responses^5^. Cytometric gating of CD56^+^ CD3^−^ cells allows relatively specific identification of circulating blood NK cells. The intensity of CD56 reflects cell functionality with CD56^bright^ NK cells showing superior responses to soluble factors whereas CD56^dim^ NK cells are geared towards recognizing cell surface-bound factors^6^. NK cell function is governed by a large number of activating and inhibitory signals. For example, NKG2D is a surface activating receptor on NK and T cells that recognizes pathogen-associated molecular patterns, whereas Killer Immunoglobulin-like Receptors (KIR) recognize host HLA molecules, and can represent major inhibitory receptors^5,7^ depending on immune education events with self-HLA ligands.

Many cytokines and chemokines detected at elevated levels immediately after rVSV-ZEBOV immunization (IL-1ra, IL-6, IL-10, TNF-α, MCP-1/CCL2, and MIP-1β/CCL4)^8,9^ can also influence the maturation/trafficking of NK cells and may be secreted by them^5,10–12^. Early rVSV-ZEBOV responses (including IL-15 and type I interferon) were hypothesized to activate NK cells^4^, and additionally, certain post-vaccination NK cell phenotypes have been shown to correlate with long-term vaccine-specific Ab responses^8^. This latter study focused on how very early immune responses to rVSV-ZEBOV could shape immune memory, whereas potential augmentation of immediate antiviral protection via vaccine-induced innate immunity was not evaluated. Therefore, to explore the potential contribution of NK cells to early protection from Ebolavirus, we investigated the changes in NK cell numbers, phenotypes and modulated gene-transcripts in a subset (*n* = 22) of Geneva vaccinees (NCT02287480)^3,13^, after an rVSV-ZEBOV high dose (HD)−i.e. currently used in Africa, compared to a low dose (LD), occurring in the first week after vaccination.

## Results

**Fig. 1:**
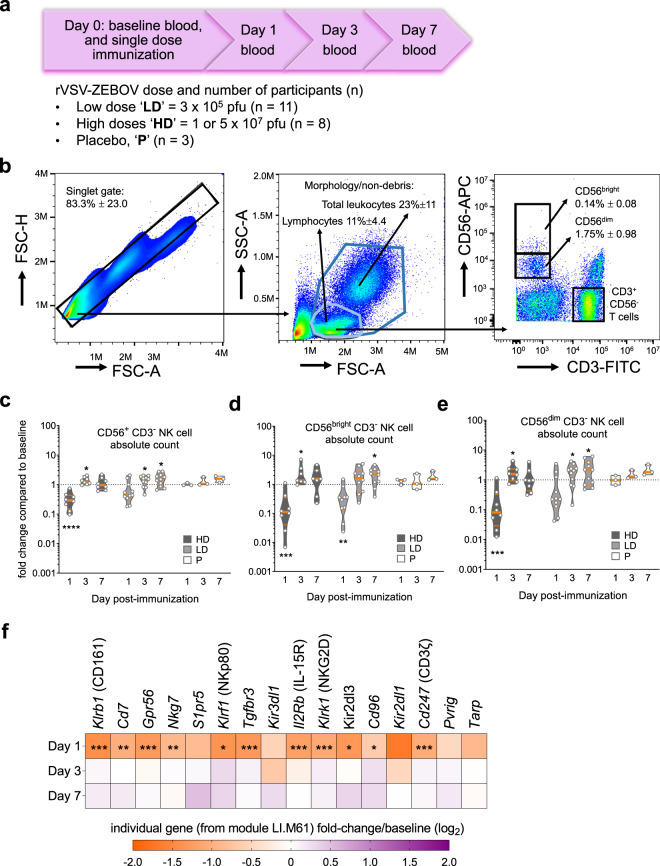
rVSV-ZEBOV modulation of CD56^+^ lymphocyte subsets. **a** Study summary of volunteers injected with high (HD) or low dose (LD) of rVSV-ZEBOV, or placebo (P). **b** Dot plots depict representative HD vaccinee PBMCs at baseline, gate values indicate cohort-wide % of pre-gated singlet, non-debris, leukocytes ± SD, followed by positive selection of either total leukocytes (dark blue gate), or non-granulocyte “lymphocyte”-like (light blue gate) followed by CD56^bright^ or CD56^dim^ NK cell selection. **c**−**e** Absolute number of **c** total NK cells (combined CD56^bright^ and CD56^dim^ NK cells), **d** CD56^bright^ NK and **e** CD56^dim^ NK cells. Violin plot symbols (open circles) show the intra-vaccinee net ratio compared to the baseline values (dashed lines), as well as median (thick red line), and interquartile (thin red line) values, shaded vaccine doses; (black squares) HD, (gray squares) LD, (open squares) placebo, and enclosed shaded horizontal width indicates the probability of obtaining the corresponding *y-*axis value. A one sample *t* test compared days 1, 3, or 7 to baseline ratio values with an expected value of 1. **f** Targeted sequencing was used to determine the average day 1, 3, and 7 to baseline fold change for genes in BTM LI.M61.0 “enriched in NK cells (II)” in HD vaccinees, indicated by the color scale, and compared using the edgeR glmtreat command with Benjamini−Hochberg multiple testing corrections. *P < 0.05, **P < 0.01, ***P < 0.001, ****P < 0.0001.

Repeated blood draws (Fig. 1a) were used to monitor the proportions and phenotype of major NK cell subsets (Fig. 1b) in fresh PBMCs following rVSV-ZEBOV administration. Baseline variability of absolute numbers and percentages of NK cells (Supplementary Fig. 1) were observed in the cohort; hence, we calculated the ratios of any changes in NK cells at the intra-participant level. On day 1, HD vaccination induced pronounced and transient reductions in total NK cell counts and percentages (Fig. 1c and Supplementary Fig. 1), including both CD56^bright^ and CD56^dim^ subpopulations (Fig. 1d, e). In contrast, LD vaccination only significantly reduced CD56^bright^ NK cell numbers (Fig. 1d), which represent a minority of circulating NK cells. A rebound or expansion was seen after HD/LD vaccination, with higher numbers (Fig. 1c−e) and percentages (Supplementary Fig. 1) of NK cells on days 3 or 7 compared to baseline. Unsupervised blood transcription module (BTM) analysis of HD vaccinees identified gene module LI.M61.0 “enriched in NK cells (II)”, as the most downregulated of all 346 BTMs on day 1 (manuscript in press), returning to baseline levels by day 3 (Supplementary Fig. 1). Many NK-cell-related genes from this BTM, including indicators of activation (*Klrk1*; encoding NKG2D), potential inhibition (*Kir2DL3*), maturation (*Nkp80*), migration (*S1pr5*) and cytokine responsiveness (*Tgfbr3*; encoding IL2RB), were downregulated on day 1 compared to day 0 (Fig. 1f). Therefore, cytometry and transcriptomics suggested early and transient total NK cell reductions in the blood after HD vaccination.

**Fig. 2:**
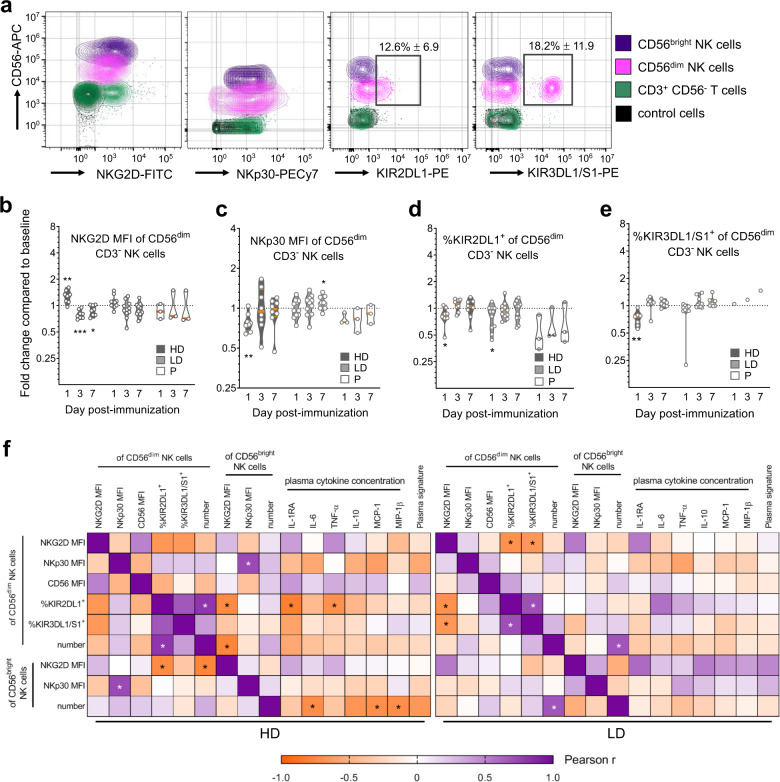
Modulation of NK cell-related surface markers and soluble proteins within the first week of rVSV-ZEBOV vaccination. **a** Cytometry contour plots of the indicated cell subsets, colored as indicated, from a representative HD vaccinee at baseline showing vaccine-modulated surface markers after gating as described in Fig. 1a. The indicated KIR^+^ subset gates are shown as open rectangles with values of cohort-wide % of total NK cells ± SD, and control cells represent total leukocytes from unstained samples. **b**−**e** Fold change analysis of CD56^dim^ NK cells compared to the baseline, depicted and compared with statistics as described for the violin plots in Fig. 1, using the geometric MFI of surface markers **b** NKG2D, **c** NKp30, or percentages of **d** KIR2DL1^+^ or **e** KIR3DL1/S1^+^ cells within the CD56^dim^ NK cell gate. **f** Two-tailed Pearson analysis comparing the indicated NK cell phenotypic parameters with plasma cytokine concentrations from matched vaccinees, all on day 1 post vaccination. Pearson *r* values were calculated using a 95% CI. *P < 0.05, **P < 0.01, ***P < 0.001.

Phenotypic changes in NK cells subsets were investigated, with all significantly modulated markers shown in Fig. 2a and Supplementary Fig. 1, and nonmodulated markers listed in the Methods. A high vaccine dose increased the expression of two activation markers; NKG2D (Fig. 2b) and CD56 (Supplementary Fig. 1) in CD56^dim^ NK cells, and reduced NKp30 expression in both CD56^bright^ and CD56^dim^ subsets (Fig. 2c, Supplementary Fig. 1). The proportion of KIR2DL1^+^ CD56^dim^ NK cells was significantly reduced in HD and LD vaccinees (Fig. 2d), as well as the KIR3DL1^+^KIR3DS1^+^ (CD158e1/e2^+^) subset after HD vaccination (Fig. 2e). Most phenotypic changes returned to baseline levels by days 3 or 7 (Fig. 2b−e), during the re-expansion of total CD56^+^ cell numbers and percentages (Fig. 1c, Supplementary Fig. 1). The few changes persisting beyond day 1 included NKG2D downregulation in HD vaccinees (Fig. 2b, Supplementary Fig. 1), and increased NKp30 expression in CD56^dim^ NK cells after LD vaccination (Fig. 2c). Overall, these phenotypic changes indicate the significant activation of CD56^dim^ NK cells in HD, and to a lesser extent in LD rVSV-ZEBOV vaccinees.

To determine whether vaccine-modulated NK-cell-related parameters were related to each other, to previously identified individual cytokine levels^9^ or to our previously described rVSV-ZEBOV plasma cytokine signature^9^, we performed Pearson comparisons between pairs of parameters from day 1 post immunization, including dose-based data stratification (HD, left panel; LD, right panel; Fig. 2f, Pearson *r* and *P* values in Supplementary Tables 1–4). The plasma signature represents a combined score of 6 cytokines/chemokines identified using the entire Geneva cohort^9^. A greater number of significant correlations existed in HD than LD vaccinees, again emphasizing the dose-related effects of rVSV-ZEBOV. In HD vaccinees, negative correlations existed between the proportion of KIR2DL1 receptor-expressing CD56^dim^ NK cells and activating NKG2D receptor expression levels in CD56^bright^ NK cells, and also with levels of NK activating IL-1ra^10^ or TNF-α^12^ plasma levels (left panel, Fig. 2f). Strikingly, the plasma level of three cytokines, IL-6, MCP-1, and MIP-1β, were inversely correlated with the number of circulating CD56^bright^ “cytokine-responsive” NK cells. A nonsignificant negative correlation trend existed between the plasma cytokine signature in HD vaccinees and the numbers of cytokine-responsive CD56^bright^ NK cells (*r* = −0.66, *P* = 0.076; left panel, Fig. 2g), suggesting enhanced CD56^bright^ NK cell efflux from the blood in vaccinees with higher combined plasma cytokine concentrations.

## Discussion

This study demonstrates that the dose of rVSV-ZEBOV impacts circulating NK cell responses because heightened activation (increased NKG2D^5^ and CD56^6^), depletion of NK cells and many inverse correlations between plasma cytokine levels and percentages of potentially inhibitory KIR^+^ CD56^dim^ or numbers of cytokine-responsive CD56^bright^ NK cells were only detected after HD vaccination.

Circulating NK cells are likely to be activated by many of the cytokines induced by rVSV-ZEBOV^4,8,9^. Within the top 15 most upregulated BTMs, three were for cytokine families (manuscript in preparation) previously defined as strong NK cell activators, namely type I IFNs^14^, IL-15^15^, and TNF-α^12^. The striking NK cell depletion observed may be due to activation-induced cell death or potential margination, i.e. adherence of the activated cells to vessel walls. However, we postulate that rVSV-ZEBOV-activated NK cells emigrate from the blood towards vaccine replication sites, including injection sites where many innate cell subsets accumulate^16^. By day 3, the blood NK cell compartment was replenished with CD56^+^ cells expressing baseline levels of nearly all investigated markers except NKG2D, which could indicate an influx of NK cells of different developmental stages^5^ or VSV-mediated NKG2D modulation^17^. NKp30 modulation was also detected in our study, but this can lead to either IFN-γ or IL-10 induction, such that the consequence of this modulation cannot be inferred^18^. Together, these data led us to postulate that activated NK cell subsets in HD rVSV-ZEBOV vaccinees may contribute to the early control and clearance of Ebolavirus.

A limitation of our study is the small number of subjects that could be studied using fresh blood during a rapidly enrolling clinical study, and the absence of RNA analysis at the single-cell level. This latter omission precluded in-depth lineage analysis of CD56^+^ cells, and restricted the investigation of how changes in the proportions of other leukocyte subsets could affect bulk transcriptomic results. Nonetheless, the massive cellular efflux and elevated activation markers of CD56^+^ cells measured by flow cytometry provides a rationale to further decipher the role of NK cells after rVSV-ZEBOV vaccination.

## Methods

Healthy adults (*n* = 115) participating in clinical trial NCT02287480^3^ were injected with rVSV-ZEBOV HD (1 or 5 × 10^7^ plaque forming units; pfu), LD (3 × 10^5^ pfu) or saline placebo (P). The study was approved and overseen by the Ethics Commission of the Canton of Geneva, Switzerland; World Health Organization’s Ethics Review Committee, and complied with all relevant regulations for work with human participants. Written informed consent was obtained from all participants. Twenty-two participants were randomly selected for in-depth characterization of circulating CD56^+^ cells at the single-cell level by flow cytometry. When unblinded, these included 8, 11, and 3 participants in the HD, LD and placebo groups respectively. Calculation of absolute numbers, surface marker fluorescence intensity, and ratios are described in the Supplementary Methods.

Ion AmpliSeq™ Transcriptome Human Gene Expression Kits (Life Technologies) were used to sequence total RNA on an Ion Proton platform, and analyzed using the edgeR package. Blood draws, sample preparation, flow cytometry or transcriptomic analyses, and statistics are described in detail in the Supplementary Methods. Transcriptomic^19^ and flow cytometry data^20^ were uploaded to online databases.

### Reporting summary

Further information on research design is available in the Nature Research Reporting Summary linked to this article.

## Supplementary information

Supplementary Information

Reporting Summary

## Data Availability

Data that support the findings of this study are available at 10.5281/zenodo.3415147 (transcriptomics)^19^, and 10.6084/m9.figshare.11886678 (flow cytometry and additional data)^20^.
