# Author Correction: Rapid dose-dependent Natural Killer (NK) cell modulation and cytokine responses following human rVSV-ZEBOV Ebolavirus vaccination

**DOI:** 10.1038/s41541-020-00244-w

**Published:** 2020-10-06

**Authors:** David Pejoski, Casimir de Rham, Paola Martinez-Murillo, Francesco Santoro, Floriane Auderset, Donata Medaglini, Gianni Pozzi, Maria Vono, Paul-Henri Lambert, Angela Huttner, Mariëlle C. Haks, Tom H. M. Ottenhoff, Jean Villard, Claire-Anne Siegrist, the VEBCON Consortium, Marylyn M. Addo, Selidji Todagbe Agnandji, Stephan Becker, Philip Bejon, Jessica S. Brosnahan, Patricia Fast, Angela Huttner, Verena Krähling, Marie-Paule Kieny, Peter G. Kremsner, Sanjeev Krishna, Olivier Lapujade, Vasee Moorthy, Patricia Njuguna, Barbara Savarese, Claire-Anne Siegrist, VSV-EBOVAC Consortium, Selidji Todagbe Agnandji, Rafi Ahmed, Jenna Anderson, Floriane Auderset, Philip Bejon, Luisa Borgianni, Jessica S. Brosnahan, Annalisa Ciabattini, Olivier Engler, Mariëlle C. Haks, Ali M. Harandi, Donald Gray Heppner, Alice Gerlini, Angela Huttner, Peter G. Kremsner, Paola Martinez-Murillo, Donata Medaglini, Thomas Monath, Francis Ndungu, Patricia Njuguna, Tom H. M. Ottenhoff, Mark Page, David Pejoski, Gianni Pozzi, Francesco Santoro, Claire-Anne Siegrist, VSV-EBOPLUS Consortium, Selidji Todagbe Agnandji, Jenna Anderson, Floriane Auderset, Luisa Borgianni, Annalisa Ciabattini, Sheri Dubey, Olivier Engler, José F. Fernandes, Mariëlle C. Haks, Ali M. Harandi, Alice Gerlini, Angela Huttner, Peter G. Kremsner, Paola Martinez-Murillo, Donata Medaglini, Thomas Monath, Helder Nakaya, Fiona O’Rourke, Tom H. M. Ottenhoff, David Pejoski, Gianni Pozzi, Sylvia Rothenberger, Francesco Santoro, Claire-Anne Siegrist

**Affiliations:** 1 Department of Pathology and Immunology, Faculty of Medicine, University of Geneva, Geneva, Switzerland; 2 World Health Organization Collaborating Centre for Vaccine Immunology, Faculty of Medicine, University of Geneva, Geneva, Switzerland; 3 Immunology and Transplantation Unit, Geneva University Hospitals, Geneva, Switzerland; 4 Department of Medical Biotechnologies, University of Siena, Siena, Italy; 5 Infectious Diseases Service, Geneva University Hospitals, Geneva, Switzerland; 6 Department of Infectious Diseases, Leiden University Medical Center, Leiden, The Netherlands; 7 University Medical Center Hamburg, Hamburg, Germany; 8 Centre de Recherches Médicales de Lambaréné, Lambaréné, Gabon; 9 Kenya Medical Research Institute, Kilifi, Kenya; 10 Institut für Tropenmedizin, Universitätsklinikum Tübingen, Tübingen, Germany; 11 World Health Organization, Geneva, Switzerland; 12 Institute of Virology, Marburg, Germany; 13 St George’s University of London, London, UK; 14 Emory Vaccine Centre, Atlanta, GA USA; 15 University of Gothenburg, Gothenburg, Sweden; 16 Sclavo Vaccines Association, Siena, Italy; 17 Spiez Laboratory, Spiez, Switzerland; 18 Crozet BioPharma, Devens, MA USA; 19 Newlink Genetics, Ames, IA USA; 20 Centre for Tropical Medicine and Global Health, Oxford University, Oxford, UK; 21 National Institute for Biological Standards and Control, Hertfordshire, UK; 22 Merck Research Laboratories, Merck and Co., Kenilworth, NJ USA; 23 Department of Clinical Analyses and Toxicology, University of Sao Paolo, Sao Paolo, Brazil

**Keywords:** Innate immunity, Vaccines, Live attenuated vaccines

Correction to: *npj Vaccines* 10.1038/s41541-020-0179-4, published online 14 April 2020

The original version of this Article contained an error in the spelling of a member of the VSV-EBOVAC and VSV-EBOPLUS Consortia, Ali M. Harandi, which was incorrectly given as Ali Harandi. This has now been corrected in both the PDF and HTML versions of the Article.

