# Supplementary material for: Rapid dose-dependent Natural Killer (NK) cell modulation and cytokine responses following human rVSV-ZEBOV Ebolavirus vaccination
Source: NPJ Vaccines. 2020 Apr 14;5:32. doi: 10.1038/s41541-020-0179-4 (PMC7156503; doi:10.1038/s41541-020-0179-4)
Supplement: Supplementary file 1 — Supplementary Information [file 41541_2020_179_MOESM1_ESM.pdf]

Supplementary Figure 1

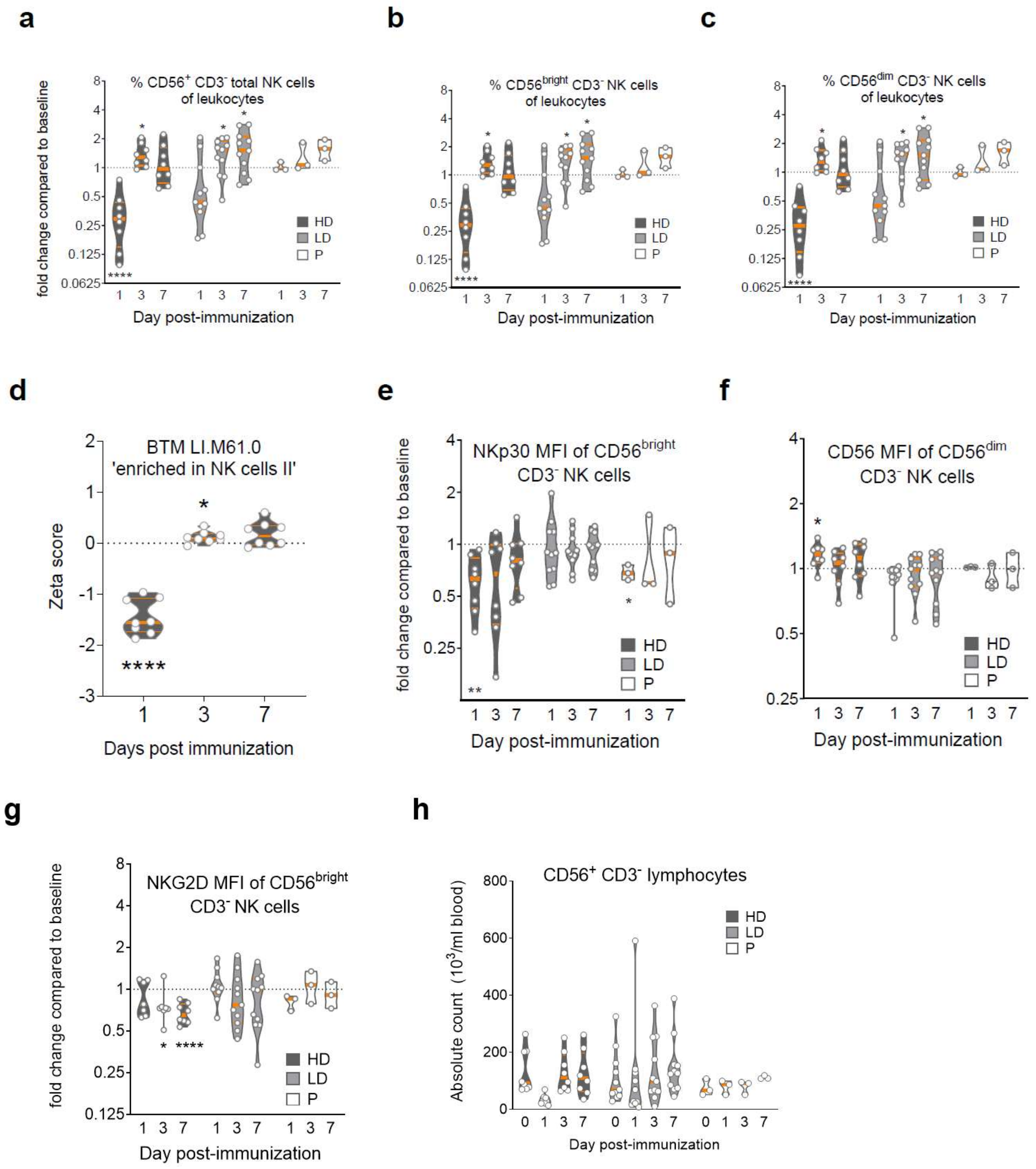

Supplementary Figure 1. **rVSV-ZEBOV modulation of percentages and phenotype of blood $CD56^+$ cell subsets**. (**a-c, e-f**) Circulating PBMC subsets from vaccinees described in Fig. 1 were stained and flow cytometry plots show (**a**) the percentages of total NK cells (combined $CD56^{bright}$ and $CD56^{dim}$ populations), **b** or $CD56^{bright}$, or (**c**) $CD56^{dim}$, calculated from the total leukocyte gate, see Fig. 1 for violin plot description and gating strategy. **d** BTM analysis showing changes in module LI.M61.0 compared to the net baseline zeta score for each subject on each study day. **e** NKp30 MFI of $CD56^{bright}$ NK cells, or (**f**) CD56 or (**g**) NKG2D of $CD56^{bright}$ NK cells were quantified in vaccinees at baseline and Day 1 post HD or LD vaccination. Statistics comprised of a one sample t test as described in Fig. 1, using an expected value of 1 for cytometry fold-change analysis, or 0 for gene module enrichment. *P < 0.05, **P < 0.01, ***P < 0.001, ****P < 0.0001. (h) shows the absolute numbers of total NK cells at the indicated time post-immunization.

SUPPLEMENTARY TABLES

Supplementary Table 1. R values of Pearson Correlations for day 1 post HD-immunization parameters

		NKG2D MFI	NKp30 MFI	CD56 MFI	% $KIR2DL1^+$	% $KIR3DL1/S1^+$	Number	NKG2D MFI	NKp30 MFI	Number	IL-1RA	IL-6	TNF-α	IL-10	MCP-1	MIP-1β	Combined plasma signature score
		of $CD56^{dim}$ NK cells						of $CD56^{bright}$ NK cells			plasma cytokine concentration						
NKG2D MFI	of $CD56^{dim}$ NK cells	1.000	-0.302	0.500	-0.659	-0.650	-0.390	0.275	-0.344	0.110	0.388	-0.029	0.458	0.125	-0.147	-0.425	-0.178
NKp30 MFI	of $CD56^{dim}$ NK cells	-0.302	1.000	-0.470	0.236	0.214	0.229	-0.002	0.727	0.256	-0.431	-0.553	-0.017	-0.510	-0.607	-0.226	-0.546
CD56 MFI	of $CD56^{dim}$ NK cells	0.500	-0.470	1.000	-0.492	-0.087	-0.417	0.080	-0.393	0.205	0.412	0.173	0.336	0.248	0.244	-0.002	0.277
%$KIR2DL1^+$	of $CD56^{dim}$ NK cells	-0.659	0.236	-0.492	1.000	0.673	0.809	-0.730	0.015	0.151	-0.803	-0.435	-0.695	-0.460	-0.278	-0.145	-0.240
%$KIR3DL1/S1^+$	of $CD56^{dim}$ NK cells	-0.650	0.214	-0.087	0.673	1.000	0.394	-0.355	-0.112	-0.167	-0.584	-0.172	-0.272	-0.207	0.029	0.166	0.094
number	of $CD56^{dim}$ NK cells	-0.390	0.229	-0.417	0.809	0.394	1.000	-0.767	-0.181	0.323	-0.551	-0.396	-0.326	-0.293	-0.334	-0.264	-0.243
NKG2D MFI	of $CD56^{bright}$ NK cells	0.275	-0.002	0.080	-0.730	-0.355	-0.767	1.000	0.112	-0.204	0.348	0.1713	0.326	0.055	0.041	0.018	-0.036
NKp30 MFI	of $CD56^{bright}$ NK cells	-0.344	0.727	-0.393	0.015	-0.112	-0.181	0.112	1.000	0.147	-0.108	-0.2123	-0.177	-0.292	-0.269	0.096	-0.281
number	of $CD56^{bright}$ NK cells	0.110	0.256	0.205	0.151	-0.167	0.323	-0.204	0.147	1.000	-0.3517	-0.7180	-0.359	-0.665	-0.708	-0.713	-0.658
IL-1RA	plasma concentration	0.388	-0.431	0.412	-0.803	-0.584	-0.551	0.348	-0.108	-0.351	1.0000	0.8177	0.740	0.856	0.692	0.570	0.686
IL-6	plasma concentration	-0.029	-0.553	0.173	-0.435	-0.172	-0.396	0.171	-0.212	-0.718	0.8177	1.0000	0.516	0.957	0.966	0.878	0.945
TNF-α	plasma concentration	0.458	-0.017	0.336	-0.695	-0.272	-0.326	0.326	-0.177	-0.359	0.7400	0.5163	1.000	0.677	0.379	0.343	0.440
IL-10	plasma concentration	0.125	-0.510	0.248	-0.460	-0.207	-0.293	0.055	-0.292	-0.665	0.8560	0.9577	0.677	1.000	0.906	0.799	0.917
MCP-1	plasma concentration	-0.147	-0.607	0.244	-0.278	0.029	-0.334	0.041	-0.269	-0.708	0.6924	0.9662	0.379	0.906	1.000	0.904	0.986
MIP-1β	plasma concentration	-0.425	-0.226	-0.002	-0.145	0.166	-0.264	0.018	0.0963	-0.713	0.5701	0.8783	0.343	0.799	0.904	1.000	0.910
Combined plasma signature score		-0.178	-0.546	0.277	-0.240	0.094	-0.243	-0.036	-0.2817	-0.658	0.6869	0.9458	0.440	0.917	0.986	0.910	1.000

Supplementary Table 2. R values of Pearson Correlations for day 1 post LD-immunization parameters

		NKG2D MFI	NKp30 MFI	CD56 MFI	% KIR2DL1^{+}	% KIR3DL1/S1^{+}	Number	NKG2D MFI	NKp30 MFI	Number	IL-1RA	IL-6	TNF-α	IL-10	MCP-1	MIP-1β	
		of CD56^{dim} NK cells						of CD56^{bright} NK cells			plasma cytokine concentration						Combined plasma signature score
NKG2D MFI	of CD56^{dim} NK cells	1.000	0.180	-0.155	-0.691	-0.655	-0.205	0.531	-0.186	-0.270	0.535	-0.268	-0.052	0.002	-0.045	-0.090	0.060
NKp30 MFI	of CD56^{dim} NK cells	0.180	1.000	0.261	-0.026	-0.209	0.215	0.128	-0.226	-0.033	-0.331	-0.392	-0.575	-0.150	-0.334	-0.294	-0.071
CD56 MFI	of CD56^{dim} NK cells	-0.155	0.261	1.000	0.373	0.128	-0.145	0.066	-0.137	0.216	0.057	0.191	0.045	-0.058	-0.064	-0.194	-0.135
%KIR2DL1^{+}	of CD56^{dim} NK cells	-0.691	-0.026	0.373	1.000	0.704	0.006	-0.007	0.148	-0.154	-0.181	0.569	0.352	0.214	0.142	0.094	0.035
%KIR3DL1/S1^{+}	of CD56^{dim} NK cells	-0.655	-0.209	0.128	0.704	1.000	0.295	-0.387	0.159	0.188	-0.290	0.169	0.113	-0.135	-0.321	-0.200	-0.133
number	of CD56^{dim} NK cells	-0.205	0.215	-0.145	0.006	0.295	1.000	-0.226	-0.042	0.685	-0.334	-0.290	-0.380	-0.270	-0.319	-0.155	-0.187
NKG2D MFI	of CD56^{bright} NK cells	0.531	0.128	0.066	-0.007	-0.387	-0.226	1.000	0.348	-0.514	0.596	0.246	0.203	0.522	0.426	0.389	0.478
NKp30 MFI	of CD56^{bright} NK cells	-0.186	-0.226	-0.137	0.148	0.159	-0.042	0.348	1.000	-0.289	-0.142	-0.160	0.369	0.243	0.130	0.143	0.294
number	of CD56^{bright} NK cells	-0.270	-0.033	0.216	-0.154	0.188	0.685	-0.514	-0.289	1.000	-0.124	-0.239	-0.399	-0.286	-0.164	-0.091	-0.185
IL-1RA	plasma concentration	0.535	-0.331	0.057	-0.181	-0.290	-0.334	0.596	-0.142	-0.124	1.000	0.456	0.154	0.595	0.569	0.583	0.570
IL-6	plasma concentration	-0.268	-0.392	0.191	0.569	0.169	-0.290	0.246	-0.160	-0.239	0.456	1.000	0.358	0.545	0.534	0.571	0.320
TNF-α	plasma concentration	-0.052	-0.575	0.045	0.352	0.113	-0.380	0.203	0.369	-0.399	0.154	0.358	1.000	0.183	0.344	0.046	-0.065
IL-10	plasma concentration	0.002	-0.150	-0.058	0.214	-0.135	-0.270	0.522	0.243	-0.286	0.595	0.545	0.183	1.000	0.802	0.914	0.941
MCP-1	plasma concentration	-0.045	-0.334	-0.064	0.142	-0.321	-0.319	0.426	0.130	-0.164	0.569	0.534	0.344	0.802	1.000	0.869	0.670
MIP-1β	plasma concentration	-0.090	-0.294	-0.194	0.094	-0.200	-0.155	0.389	0.143	-0.091	0.583	0.571	0.046	0.914	0.869	1.000	0.877
Combined plasma signature score		0.060	-0.071	-0.135	0.035	-0.133	-0.187	0.478	0.294	-0.185	0.570	0.320	-0.065	0.941	0.670	0.877	1.000

Supplementary Table 3. P values of Pearson Correlations for day 1 post HD-immunization parameters

		NKG2D MFI	NKp30 MFI	CD56 MFI	% $KIR2DL1^{+}$	% $KIR3DL1/S1^{+}$	Number	NKG2D MFI	NKp30 MFI	Number	IL-1RA	IL-6	TNF-α	IL-10	MCP-1	MIP-1β	Combined plasma signature score
		of $CD56^{dim}$ NK cells						of $CD56^{bright}$ NK cells			plasma cytokine concentration						
NKG2D MFI	of $CD56^{dim}$ NK cells	-	0.466	0.206	0.075	0.081	0.339	0.509	0.404	0.795	0.342	0.944	0.253	0.767	0.726	0.293	0.671
NKp30 MFI	of $CD56^{dim}$ NK cells	0.466	-	0.239	0.572	0.610	0.585	0.995	0.040	0.539	0.285	0.155	0.967	0.196	0.110	0.590	0.161
CD56 MFI	of $CD56^{dim}$ NK cells	0.206	0.239	-	0.215	0.837	0.303	0.850	0.334	0.625	0.310	0.681	0.414	0.552	0.560	0.995	0.506
%$KIR2DL1^{+}$	of $CD56^{dim}$ NK cells	0.075	0.572	0.215	-	0.067	0.015	0.039	0.970	0.720	0.016	0.280	0.055	0.250	0.504	0.731	0.565
%$KIR3DL1/S1^{+}$	of $CD56^{dim}$ NK cells	0.081	0.610	0.837	0.067	-	0.333	0.387	0.790	0.691	0.128	0.682	0.513	0.621	0.945	0.693	0.823
number	of $CD56^{dim}$ NK cells	0.339	0.585	0.303	0.015	0.333	-	0.026	0.666	0.435	0.156	0.331	0.429	0.480	0.417	0.526	0.561
NKG2D MFI	of $CD56^{bright}$ NK cells	0.509	0.995	0.850	0.039	0.387	0.026	-	0.790	0.627	0.397	0.685	0.429	0.895	0.922	0.965	0.931
NKp30 MFI	of $CD56^{bright}$ NK cells	0.404	0.040	0.334	0.970	0.790	0.666	0.790	-	0.727	0.797	0.613	0.674	0.481	0.518	0.820	0.499
number	of $CD56^{bright}$ NK cells	0.795	0.539	0.625	0.720	0.691	0.435	0.627	0.727	-	0.392	0.044	0.381	0.071	0.049	0.046	0.075
IL-1RA	plasma concentration	0.342	0.285	0.310	0.016	0.128	0.156	0.397	0.797	0.392	-	0.013	0.035	0.006	0.057	0.140	0.059
IL-6	plasma concentration	0.944	0.155	0.681	0.280	0.682	0.331	0.685	0.613	0.044	0.013	-	0.190	0.001	0.001	0.004	0.001
TNF-α	plasma concentration	0.253	0.967	0.414	0.055	0.513	0.429	0.429	0.674	0.381	0.035	0.190	-	0.065	0.353	0.405	0.275
IL-10	plasma concentration	0.767	0.196	0.552	0.250	0.621	0.480	0.895	0.481	0.071	0.006	0.001	0.065	-	0.001	0.017	0.001
MCP-1	plasma concentration	0.726	0.110	0.560	0.504	0.945	0.417	0.922	0.518	0.049	0.057	0.001	0.353	0.001	-	0.002	0.001
MIP-1β	plasma concentration	0.293	0.590	0.995	0.731	0.693	0.526	0.965	0.820	0.046	0.140	0.004	0.405	0.017	0.0020	-	0.002
Combined plasma signature score		0.671	0.161	0.506	0.565	0.823	0.561	0.931	0.499	0.075	0.059	0.001	0.275	0.001	0.0001	0.002	-

Supplementary Table 4. P values of Pearson Correlations for day 1 post LD-immunization parameters

		NKG2D MFI	NKp30 MFI	CD56 MFI	% KIR2DL1$^+$	% KIR3DL1/S1$^+$	Number	NKG2D MFI	NKp30 MFI	Number	IL-1RA	IL-6	TNF-α	IL-10	MCP-1	MIP-1β	Combined plasma signature score
		of CD56^{dim} NK cells						of CD56^{bright} NK cells			plasma cytokine concentration						
NKG2D MFI	of CD56^{dim} NK cells	-	0.596	0.648	0.018	0.028	0.544	0.092	0.582	0.422	0.089	0.424	0.877	0.996	0.894	0.790	0.859
NKp30 MFI	of CD56^{dim} NK cells	0.596	-	0.437	0.939	0.537	0.525	0.707	0.504	0.922	0.318	0.232	0.069	0.658	0.314	0.379	0.835
CD56 MFI	of CD56^{dim} NK cells	0.648	0.437	-	0.258	0.706	0.669	0.845	0.686	0.521	0.867	0.573	0.894	0.863	0.850	0.565	0.691
%KIR2DL1$^+$	of CD56^{dim} NK cells	0.018	0.939	0.258	-	0.015	0.985	0.983	0.662	0.650	0.593	0.067	0.288	0.526	0.677	0.782	0.918
%KIR3DL1/S1$^+$	of CD56^{dim} NK cells	0.028	0.537	0.706	0.015	-	0.378	0.239	0.640	0.577	0.386	0.618	0.739	0.691	0.335	0.554	0.695
number	of CD56^{dim} NK cells	0.544	0.525	0.669	0.985	0.378	-	0.502	0.900	0.019	0.314	0.386	0.248	0.421	0.338	0.647	0.581
NKG2D MFI	of CD56^{bright} NK cells	0.092	0.707	0.845	0.983	0.239	0.502	-	0.293	0.105	0.053	0.465	0.549	0.098	0.191	0.236	0.136
NKp30 MFI	of CD56^{bright} NK cells	0.582	0.504	0.686	0.662	0.640	0.900	0.293	-	0.387	0.675	0.637	0.263	0.470	0.702	0.674	0.379
number	of CD56^{bright} NK cells	0.422	0.922	0.521	0.650	0.577	0.019	0.105	0.387	-	0.715	0.477	0.224	0.393	0.629	0.789	0.585
IL-1RA	plasma concentration	0.089	0.318	0.867	0.593	0.386	0.314	0.053	0.675	0.715	-	0.157	0.650	0.053	0.067	0.059	0.066
IL-6	plasma concentration	0.424	0.232	0.573	0.067	0.618	0.386	0.465	0.637	0.477	0.157	-	0.279	0.082	0.090	0.066	0.337
TNF-α	plasma concentration	0.877	0.063	0.894	0.288	0.739	0.248	0.549	0.263	0.224	0.650	0.279	-	0.590	0.298	0.892	0.849
IL-10	plasma concentration	0.996	0.658	0.863	0.526	0.691	0.421	0.098	0.470	0.393	0.053	0.082	0.590	-	0.003	0.001	0.001
MCP-1	plasma concentration	0.894	0.314	0.850	0.677	0.335	0.338	0.191	0.702	0.629	0.067	0.090	0.298	0.003	-	0.001	0.023
MIP-1β	plasma concentration	0.790	0.379	0.565	0.782	0.554	0.647	0.236	0.674	0.789	0.059	0.066	0.892	0.001	0.001	-	0.001
Combined plasma signature score		0.859	0.835	0.691	0.918	0.695	0.581	0.136	0.379	0.585	0.066	0.337	0.849	0.001	0.023	0.001	-

SUPPLEMENTARY METHODS

Human blood sampling and processing

The study was approved and overseen by the Ethics Commission of the Canton of Geneva, Switzerland; World Health Organization's Ethics Review Committee. Blood was drawn on days 0 ('baseline'), 1, 3, and 7 post-vaccination, collected for Peripheral blood mononuclear cells (PBMC) isolation, plasma, or RNA isolation (PAXgene® Blood RNA tubes (Qiagen). Samples were prepared according to routine procedures and as described previously[1].

PBMC staining and cytometry acquisition

Freshly isolated PBMCs (0.5 million per Ab panel) were incubated for 20 mins at 4°C with antibody reagents, described in full in the Reporting Statement. Abs purchased from Biolegend included: CD3-FITC , BD Pharmingen: CD3-PeCy7, KIR2DL1 CD158a-FITC, KIR3DL1 CD158e1/e2-PE, Miltenyi Biotec: CD56-APC, NKp30-biotin, NKp44-PE, NKp46-PE, CD57-APC, R&D: NKG2C-FITC, Beckman Coulter: CD56-PE, CD158b-FITC, CD158i (KARp50.3)-PE, NKG2A-PE, Serotec: NKG2D-FITC, washed, and stained with streptavidin-PeCy7 (eBioscience) if necessary. Each of the 8 staining panels contained a total of four Abs, including anti-CD3 and anti-CD56 Abs. Cells were washed and acquired immediately using an Accuri C6 cytometer. Raw cytometry data were processed and analysed with FlowJo (TreeStar), Excel (Microsoft), and GraphPad Prism. The net geometric median fluorescence intensity (gMFI) of surface marker staining was calculated after subtraction of gMFI signal obtained in similarly-gated unstained leukocyte controls. Data for surface markers that did not undergo statistically significant changes in terms of gMFI or percentages compared to baseline levels are not shown. The absolute number of (**c**) total NK cells (combined CD56 ^{bright} and CD56 ^{dim} NK cell populations), (**d**) CD56 ^{bright} NK cells, and (**e**) CD56 ^{dim} NK was calculated using the subset percentages obtained using flow cytometry and previously published total leukocyte numbers[2]. Briefly, live NK cells were defined as singlet, CD3-, CD56+ using flow cytometry. The

proportion of live NK cells of total leukocytes obtained via flow cytometry was then multiplied by the total number of leukocytes per ml blood, which was obtained using an automated whole blood cell counter at the time of sampling.

Targeted transcriptome sequencing

Sequencing libraries were prepared with the Ion AmpliSeq™ Transcriptome Human Gene Expression Kit (Life Technologies) according to manufacturer's instructions, starting from 30-50 ng of total RNA, as quantified by the RNA High Sensitivity kit on a Qubit 2.0 (Thermo Fisher). Pooled libraries were loaded on Ion PI™ Chips v3 using an Ion Chef instrument and then sequenced on the Ion Proton platform. Reads were base-called by the instrument software and aligned to human transcripts with the ampliseq plugin. Gene expression data were retrieved as .csv files and analyzed using the edgeR package [3]. Data were analyzed with the glmQLfit function, which uses generalized linear models to account for the experimental design and the quasi-likelihood *F*-tests to conduct hypothesis tests. Differential expression was then assessed for each time point against the pre-vaccination baseline using the glmtreat function with a fold-change threshold of 1.2. Raw transcriptomic data and R code used for the analysis are available at DOI: 10.5281/zenodo.3415147.

Statistics

Ratios of changes in cell surface marker MFI, the percentage of cell surface marker positive cells, or the concentration of soluble plasma proteins, were calculated by dividing the net value from Day 1, 3, or 7 with the net Day 0 baseline value. Net values were calculated as described in the assay methods above. Statistics were performed using GraphPad Prism software. A single sample t test was used to identify whether the fold change of the indicated net parameters was significantly modulated when compared using an alpha value of 0.05 and an expected value of 0 or 1 as specified in the figure legends. Two-tailed Pearson correlation matrices were generated using matched net values from individual vaccinees at day 1 post-immunization, and the r co-efficient and p values were calculated using a 95% CI. BTM activation for each time point was calculated for each subject as the average of the zeta scores of the genes in the module minus the average of genes' zeta scores at day 0.

REFERENCES

1. Huttner, A. *et al.* A dose-dependent plasma signature of the safety and immunogenicity of the rVSV-Ebola vaccine in Europe and Africa. *Sci. Transl. Med.* **9**, eaaj1701 (2017).
2. Huttner, A. *et al.* The effect of dose on the safety and immunogenicity of the VSV Ebola candidate vaccine: A randomised double-blind, placebo-controlled phase 1/2 trial. *Lancet Infect. Dis.* **15**, 1156–1166 (2015).
3. Robinson, M. D., McCarthy, D. J. & Smyth, G. K. edgeR: A Bioconductor package for differential expression analysis of digital gene expression data. *Bioinformatics* (2009). doi:10.1093/bioinformatics/btp616
